# Elegant Process of Capsuling Bottles

**Published:** 1880-02

**Authors:** 


ELEGANT PROCESS OF CAPSULING BOTTLES.—A new system of capsuling bottles has come into fashion from France. It is much more rapid than the method of affixing lead capsules, and some may think that it gives more elegant effects. The medium for forming the capsulage is a viscous volatile liquid, into which the top of the bottle is dipped, and immediately withdrawn with a slight rotary motion. It leaves a transparent capsule, and the effect is better if a label bearing a monogram or trade-mark had been previously attached to the top of the bottle. We find the following formula for the liquid, given by M. Soulan, of St. Emilion:

| | PARTS. |
|---|---|
| Yellow resin.............................. | 20 |
| Ether ...................................... | 40 |
| Collodion................................ | 60 |
| Fuchsine or other tint, q. s. | |

[*Chemist and Druggist.*

